# Heart and brain interactions: Pathophysiology and management of cardio-psycho-neurological disorders

**DOI:** 10.1007/s00059-021-05022-5

**Published:** 2021-02-05

**Authors:** Renate B. Schnabel, Gert Hasenfuß, Sylvia Buchmann, Kai G. Kahl, Stefanie Aeschbacher, Stefan Osswald, Christiane E. Angermann

**Affiliations:** 1 Universitäres Herz- und Gefäßzentrum Hamburg, Klinik und Poliklinik für Kardiologie, Universitätsklinikum Hamburg-Eppendorf, Hamburg, Germany; 2 Standort Hamburg/Kiel/Lübeck, Deutsches Zentrum für Herz-Kreislaufforschung (DZKH e. V.), Hamburg, Germany; 3 Herzzentrum, Klinik für Kardiologie und Pneumologie, Georg-August-Universität Göttingen, Göttingen, Germany; 4 Klinik für Anästhesie, operative Intensivmedizin und Schmerztherapie, Vivantes Klinikum Spandau—Berlin, Berlin, Germany; 5 Klinik für Psychiatrie, Sozialpsychiatrie und Psychotherapie, Medizinische Hochschule Hannover (MHH), Hannover, Germany; 6 Klinik für Kardiologie, Universitätsspital Basel, Basel, Switzerland; 7 Deutsches Zentrum für Herzinsuffizienz, Universität und Universitätsklinikum Würzburg, Am Schwarzenberg 15, 97078 Würzburg, Germany; 8 Zentrum für seelische Gesundheit, Klinik für Psychiatrie, Psychosomatik und Psychotherapie, Universität Würzburg, Würzburg, Germany

**Keywords:** Heart-and-brain axis, Mental health disorders, Takotsubo syndrome, Peripartum cardiomyopathy, Atrial fibrillation, Herz-Hirn-Achse, Neuropsychiatrische Erkrankungen, Takotsubo-Syndrom, Peripartum-Kardiomyopathie, Vorhofflimmern

## Abstract

Cardiovascular diseases (CVD) and mental health disorders (MHD; e.g. depression, anxiety and cognitive dysfunction) are highly prevalent and are associated with significant morbidity and mortality and impaired quality of life. Currently, possible interactions between pathophysiological mechanisms in MHD and CVD are rarely considered during the diagnostic work-up, prognostic assessment and treatment planning in patients with CVD, and research addressing bidirectional disease mechanisms in a systematic fashion is scarce. Besides some overarching pathogenetic principles shared by CVD and MHD, there are specific syndromes in which pre-existing neurological or psychiatric illness predisposes and contributes to CVD development (as in Takotsubo syndrome), or in which the distorted interplay between innate immune and central nervous systems and/or pre-existing CVD leads to secondary MHD and brain damage (as in peripartum cardiomyopathy or atrial fibrillation). Clinical manifestations and phenotypes of cardio-psycho-neurological diseases depend on the individual somatic, psychosocial, and genetic risk profile as well as on personal resilience, and differ in many respects between men and women. In this article, we provide arguments on why, in such conditions, multidisciplinary collaborations should be established to allow for more comprehensive understanding of the pathophysiology as well as appropriate and targeted diagnosis and treatment. In addition, we summarize current knowledge on the complex interactions between the cardiovascular and central nervous systems in Takotsubo syndrome and peripartum cardiomyopathy, and on the neurological and psychiatric complications of atrial fibrillation.

## Abstract (de)

Kardiovaskuläre und psychische Erkrankungen (z. B. Depression, Angststörung, Kognitionsverlust) gehören zu den häufigen Volkskrankheiten, sind jeweils mit schlechter Lebensqualität und hoher Morbidität und Mortalität assoziiert und kommen häufig gemeinsam vor. Mögliche Zusammenhänge zwischen kardiovaskulären und psychischen Gesundheitsstörungen und ihre prognostischen Implikationen bleiben bisher bei Diagnostik und Therapie kardiovaskulärer Patienten weitgehend unberücksichtigt, und systematische wissenschaftliche Untersuchungen dazu sind selten. Neben prinzipiellen systemischen Wechselwirkungen gibt es syndromale Krankheitsbilder, bei denen entweder vorbestehende neurologische oder psychiatrische Krankheiten das Risiko für die Entstehung der Herzerkrankung erhöhen oder zum Krankheitsgeschehen beitragen (wie beim Takotsubo-Syndrom) oder bei denen eine gestörte Interaktion von angeborenem Immunsystem und zentralem Nervensystem und/oder vorbestehende Herzkrankheiten zur Entstehung von sekundären psychiatrischen oder neurologischen Krankheiten führen (wie bei der Peripartum-Kardiomyopathie oder dem Vorhofflimmern). Klinische Manifestation und Phänotyp kardio-psycho-neurologischer Krankheiten hängen vom individuellen somatischen, psychosozialen und genetischen Risikoprofil und von der Resilienz des Einzelnen ab und sind bei Männern und Frauen in mancher Hinsicht unterschiedlich. In diesem Artikel wird erörtert, warum zum besseren Verständnis der Krankheitsentstehung und für die gezielte Diagnose und Behandlung solcher komplexer Erkrankungen multidisziplinäre Kooperationen wichtig sind. Außerdem wird der aktuelle Wissensstand zu den Wechselwirkungen zwischen kardialen und psychischen bzw. neurologischen Gesundheitsstörungen beim Takotsubo-Syndrom, der Peripartum-Kardiomyopathie und dem Vorhofflimmern zusammengefasst.

Mental health disorders (MHD; including depression, anxiety and/or cognitive dysfunction) and cardiovascular diseases (CVD) are both highly prevalent conditions and are among the leading causes of death worldwide [1, 2]. The current changes to worldwide population demographics mean that the prevalence of common diseases affecting the heart and the brain will increase, placing an even greater burden on healthcare systems and societies. This highlights the importance of strategies to prevent and manage these conditions.

There are considerable interactions between the heart and brain, which is partially explained by similarities in tissue properties. These include the fact that both are electrically active organs, and have high energy requirements and a low capacity for regeneration after cell death. From a pathophysiological perspective, comorbidities such as hypertension, diabetes, hyperlipidaemia and systemic inflammation as well as age and multiple stressors may impact on both cardiac and mental health conditions, and genetic or epigenetic mechanisms may affect the heart and brain in a comparable fashion. For example, autonomic dysfunction and inflammation may contribute to the increased cardiovascular mortality risk associated with depression [3], and a mutation of the ryanodine receptor sarcoplasmic reticulum calcium release channel has been shown to result in both cardiac arrhythmias and seizures [4]. Against this background, a bi-directional relationship between MHD and CVD is not surprising.

Here, we review interactions between CVD and MHD. This includes CVD and MHD in general, along with specific syndromes in which pre-existing neurological or psychiatric illnesses may predispose and contribute to CVD development (as in Takotsubo syndrome [TTS]), or in which psycho-physical stress, dysregulation of the interplay between innate immune and central nervous systems, and/or pre-existing CVD lead to secondary MHD and brain damage (as in peripartum cardiomyopathy [PPCM] and atrial fibrillation [AF]).

Awareness of the complex bidirectional disease mechanisms in these settings is growing rapidly, but knowledge gaps remain, especially regarding the management of MHD in patients with these conditions. Multidisciplinary research that crosses organ borders is essential to uncover new disease mechanisms and allow for translation of new technologies, diagnostic procedures and preventive or innovative treatments from research settings into clinical practice to provide effective management and facilitate better outcomes in patients with cardio-psycho-neurological disorders.

## Links between cardiovascular diseases and mental health: general considerations

Mental health disorders and CVD are common comorbidities [5]. The former are major causes of morbidity, mortality and poor quality of life in patients with CVD and are also independently associated with future CVD development [6, 7]. Similar to the general population, MHD (especially depression) are more prevalent in women and younger people with CVD [8]. Importantly, MHD prevalence rates vary with CVD type and severity [5]. For example, depression was reported in 20% of patients with New York Heart Association (NYHA) class I/II heart failure (HF), but in 42% of those with NYHA class III/IV HF in a study of 682 patients hospitalized with HF [9]. Furthermore, in addition to being correlated with NYHA class, depression prevalence and severity were also inversely related to quality of life [10].

### Pathophysiology

**Fig. 1:**
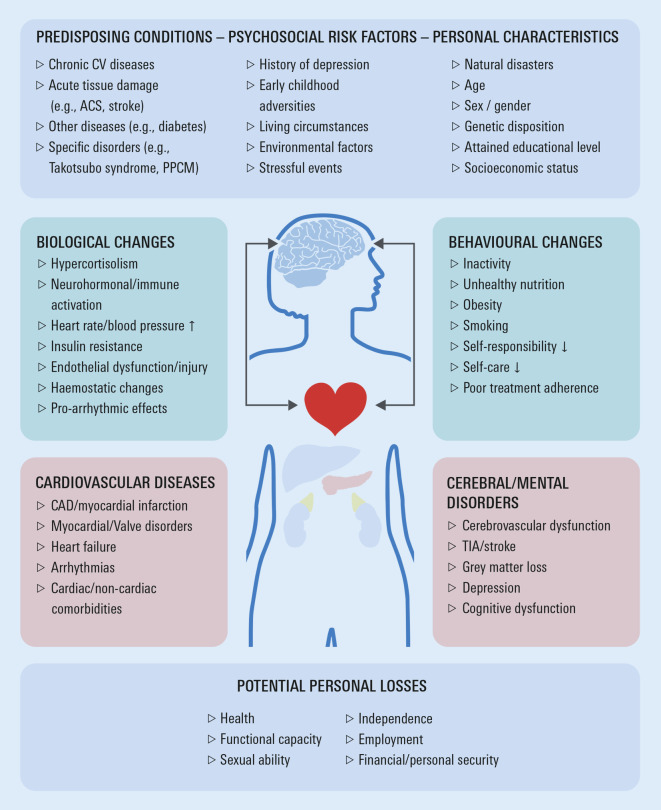
Pathophysiological links and biological and behavioural disease mechanisms potentially implicated in the development and progression of cardio-psycho-neurological disorders. *ACS* acute coronary syndrome, *PPCM* peripartum cardiomyopathy, *CV* cardiovascular, *CAD* coronary artery disease

Several studies, including large population-based analyses, report associations between MHD and incident CVD [11]. Bi-directional interrelations between major depression and CVD suggest that both diseases may share common pathophysiological pathways [12]. These include lifestyle factors (e.g. physical activity, smoking behaviour), dysfunction of endocrine systems (e.g. hypothalamus–pituitary–adrenal axis), and an imbalance of pro- and anti-inflammatory factors. Both MHD and CVD share genes involved in energy metabolism, stress system, circadian rhythm, inflammation and neurotransmission. For example, covariation of depressive symptoms and coronary disease may in part be attributable to common genetic vulnerability potentially related to inflammation, serotonin pathways or mitochondrial energy metabolism [13, 14]. Associations between a functional sequence variant of the neuropeptide S receptor‑1 gene, which regulates anxiety, and clinical outcomes and healthcare utilization in HF patients suggests the possibility that psychogenetic determinants may modulate also such endpoints [15]. Figure 1 shows a simplified schematic of key heart–brain interactions, which may induce systemic organ dysfunction and promote development and progression of different clinical phenotypes of both CVD and MHD. The pathogenetic importance of each factor varies in individual patients, and likely depends on the overall risk profile and personal resilience (i.e. the capacity to adapt swiftly and successfully in the face of physical and/or emotional challenges), plus the presence and severity of the somatic illness [16].

Similar to external stress, negative emotions might impact adversely on neurohormonal regulatory circuits. Autonomic nervous dysfunction may trigger multiple biological changes, including increased sympathetic tone, innate and adaptive immune system activation, higher circulating levels of stress hormones (e.g. cortisol and pro-inflammatory cytokines) and metabolic dysregulation [17]. Systemic inflammation may induce a procoagulatory state and endothelial dysfunction/injury. All these contribute to the development and progression of atherosclerosis, and increase the risk for arrhythmias such as AF, cardiac and cerebral clinical events and systemic disease in general [12, 18]. For example, recent evidence suggests that epicardial adipose tissue is a direct source of pro-inflammatory cytokines and could mediate various deleterious effects of systemic inflammation on cardiac structure and function, thus contributing to the pathogenesis of CVD, including calcific aortic stenosis [19]. Interestingly, transcatheter aortic valve replacement was shown to persistently improve depression and anxiety in such patients [20]. Other studies have demonstrated increased intra-abdominal and epicardial adipose tissue in individuals who are physically healthy but depressed, and in cardiac patients (e.g. congenital heart disease) with major depressive disorder [14, 21]. Lastly, MHD may influence behavioural factors such as lifestyle, self-care and adherence to evidence-based therapies [22], which further contributes to a vicious circle of declining health, functional capacity and personal socioeconomic status.

### Management

Mental health disorders can be easily overlooked or misinterpreted by physicians and patients [6] because signs and symptoms such as physical disability/fatigue, loss of interest, anhedonia and sad mood, or eating and sleep disorders may be associated with both MHD and CVD, particularly at more advanced disease stages. Systematic screening for MHD using simple validated self-report tools offers the potential for early identification and targeted management, and is widely recommended [5]. Screening for MHD in cardiologist practices and hospitals would require minimal resources and efforts, but identification of MHD might necessitate increased downstream support from mental health providers, including immediate evaluation of suspected suicidality, and thus impact significantly on routine CVD management algorithms [5]. This could be one reason why MHD screening has not been widely implemented in CVD care settings, and why MHD remain underdiagnosed and undertreated. Additional research is needed to better determine the benefit of screening CVD patients for MHD [23]. Guideline-supported screening algorithms for MHD, especially depression, and additional diagnostic steps needed after a positive screening result are detailed in the work of Jha et al. [5].

Given the close correlation between CVD severity and the prevalence and severity of MHD, especially depression [12], treatments that improve cardiovascular function should also benefit coexisting mood disorders. The recent MEMS-HF study evaluating haemodynamic-guided HF management based on remote pulmonary artery pressure (PAP) monitoring demonstrated that decreases in PAP were associated with significant, persistent remission of depressive symptoms, and that larger PAP decreases resulted in greater depression remission and quality of life improvement [24]. This is the first time that an MHD (depression) has been linked with a treatable biological variable (haemodynamic congestion). Accordingly, standard care approaches should include disease-modifying treatments for CVD and associated comorbidities as well as MHD, and integrate needs-adjusted management of stressful signs and symptoms (e.g. sleep optimization, pain management) while improving patients’ psychosocial functioning, healthcare competence and self-monitoring/empowerment [12].

Specific antidepressant and anxiolytic treatments, including pharmacotherapy, psychological or behavioural interventions and/or physical exercise, have shown disparate effects on symptoms and clinical outcomes in patients with CVD, including post-stroke depression [25, 26]. Due to their overall safety profile and effectiveness, selective serotonin re-uptake inhibitors (SSRI) have emerged as first-line therapy for depression and anxiety in patients with CVD [25]. Evidence suggests that SSRI are safe in patients with stable CVD and after myocardial infarction, and may improve depressive symptoms and possibly prognosis (overview in [5]). However, SSRI had no effect on either depression or outcomes compared with clinical management alone in patients with symptomatic HF in two large randomized trials [27, 28], and dose-dependent QTc prolongation indicates increased risk for torsade-de-pointes arrhythmias [29]. Meta-analysis data suggest that SSRI may increase mortality risk in HF patients [30]. Few data are available on the safety and efficacy of newer antidepressants such as melatonergic agonists (agomelatine) and norepinephrine and dopamine reuptake inhibitors (bupropion) in patients with CVD. Adverse clinical effects such as changes in adiposity and insulin sensitivity, or (in experimental models) endothelial nitric oxide depletion and augmentation of oxidative stress, have been observed with some of the newer antipsychotics (e.g. risperidone, olanzapine or clozapine; [31, 32]). These agents therefore seem to have the potential to cause complications that contribute to increased cardiovascular risk, necessitating careful benefit-to-risk assessment before use.

More advanced immune dysregulation as the major underlying cause of MHD, especially depression, might explain both the higher prevalence rates and the relative lack of benefit from antidepressants, particularly at more severe CVD stages. Thus, before antidepressant therapy is initiated, possible benefits should always be jointly evaluated by cardiologists and psychiatrists. Medications with anti-inflammatory properties (e.g. non-steroidal anti-inflammatories, cytokine inhibitors) have been shown to alleviate signs and symptoms of both CVD and depression [33]. In addition, recent clinical trials demonstrated that canakinumab and colchicine significantly reduce cardiovascular event rates in patients with coronary disease (reviewed by Nidorf et al. [34]). To what extent these drugs classes may also improve concurrent MHD requires further study.

## Takotsubo syndrome: neurological and psychiatric comorbidities

**Fig. 2:**
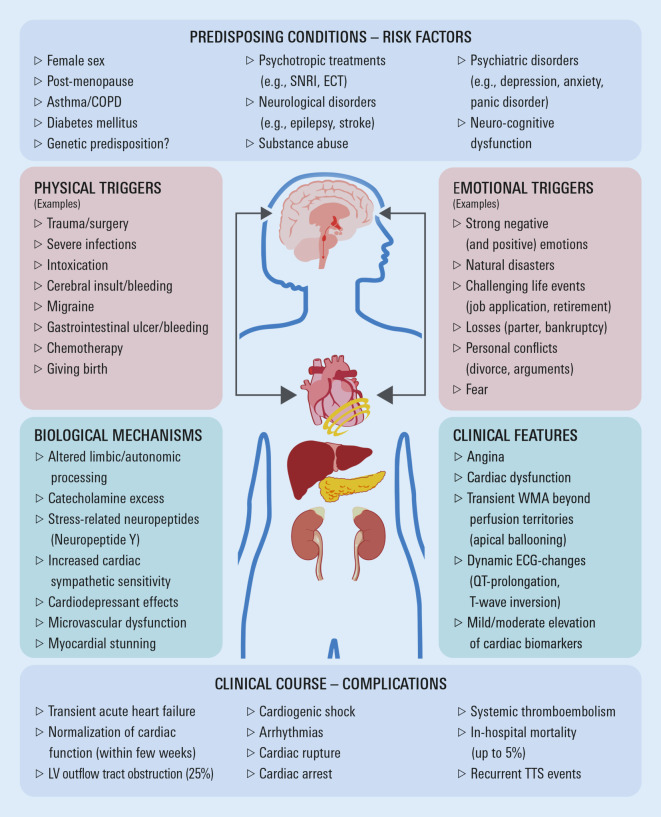
Possible pathogenic mechanisms, clinical course and complications of Takotsubo syndrome. *COPD* chronic obstructive pulmonary disease; *SNRI* serotonin-norepinephrine reuptake inhibitors, *ECT* electroconvulsive therapy, *WMA* wall motion abnormality, *ECG* electrocardiogram, *LV* left ventricular, *TTS* Takotsubo syndrome

Takotsubo syndrome (TTS), also termed “stress cardiomyopathy” or “broken heart syndrome”, represents an acute reversible form of myocardial injury characterized by transient myocardial wall motion abnormalities, and is often accompanied by symptoms of acute heart failure [35]. “Takotsubo” refers to an apical ballooning pattern of the left ventricle during systole, which is the most common cardiac wall motion abnormality in patients with TTS and resembles a Japanese octopus trap. Other parts of the myocardium may also be affected; typically, wall motion abnormalities extend beyond the perfusion territory of one vessel [35, 36]. Figure 2 shows predisposing conditions and risk factors for TTS. Typically, TTS occurs in post-menopausal women, and there is a strong association with pre-existing psychiatric or neurological illnesses or with substance abuse. Frequently, but not always, TTS is preceded by physical or emotional triggers [36]. The estimated incidence in all patients with suspected ACS is 1–2%, but TTS is likely to be underdiagnosed [37]. The International Takotsubo Registry showed that almost 90% of patients were female [38], although TTS may occasionally occur in males. The most common symptoms of TTS are acute chest pain and dyspnoea, which at first glance resemble ACS. However, in contrast to ACS, no relevant obstructive coronary lesions are seen on coronary angiography [35]. Although TTS is generally considered a benign disease, several observational studies reported rates of complications (e.g. cardiogenic shock) and clinical endpoint events that were comparable to or even worse than those of ACS. For example, in a German series of 286 patients with TTS, the 1‑year mortality rate did not differ from that in a matched group of 286 patients with ST-segment elevation myocardial infarction (STEMI), while the mortality rate after a mean 3.8 ± 2.5 years of follow-up was even higher in patients with TTS versus STEMI (24.7% vs. 15.1%, *p* = 0.02; [39]). These findings are concordant with data from the largest TTS registry to date [38], and suggest that TTS may not be as benign as previously assumed, and that patients with TTS need to be monitored long term, similar to those after STEMI.

### Pathophysiology

The pathobiology of TTS is largely unknown, but suggested mechanisms include hypoconnectivity of central brain regions leading to altered limbic and autonomic processing in response to stress [40], and excess liberation of neurohormones and neuropeptides (e.g. neuropeptide Y) in response to stressful events [36]. The increased risk of TTS development in patients with pre-existing neuro-psychiatric illnesses might be due to both an exaggerated catecholaminergic response and increased sensitivity of the myocardium to catecholamines, all of which may exert direct cardiotoxic effects and induce microvascular dysfunction and myocardial stunning [36]. In line with the concept of sympathetic activation, elevated norepinephrine levels in the coronary sinus have been found in patients with TTS, suggesting increased myocardial catecholamine release [41]. In an in vitro induced pluripotent stem cell model of TTS, enhanced β‑adrenergic signalling and higher sensitivity to catecholamine-induced toxicity were identified as mechanisms associated with TTS [42]. In addition to myocardial dysfunction and injury, all these processes lead to dynamic ECG changes and moderate elevation of cardiac biomarkers in affected patients, and are referred to as “neurogenic stunning myocardium” (see Medina de Chazal et al. for more details; [36]).

Activation of the hypothalamic–pituitary–adrenal axis might link emotional stress and clinical TTS phenomena. In addition, pathways implicating female sex hormones could be important, given that 90% of TTS patients are women. Oestrogens may modulate catecholamine-induced vasoconstriction and upregulate endothelial nitric oxide (NO) synthase. Therefore, oestrogen supplementation may attenuate glucocorticoid and catecholamine responses to mental stress in perimenopausal women [43].

### Management

**Fig. 3:**
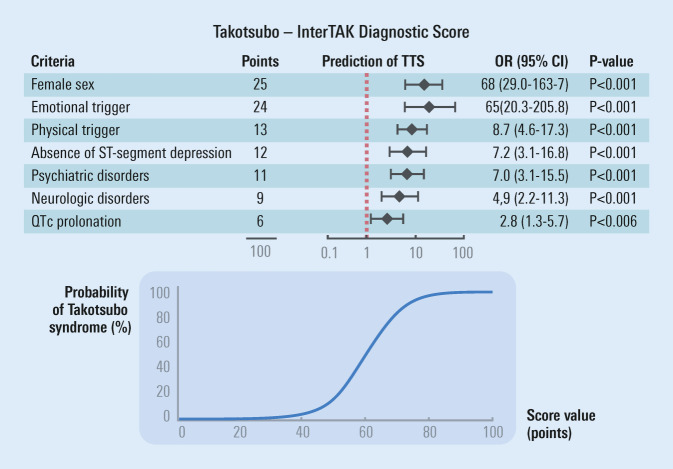
Predictors of the diagnosis of Takotsubo syndrome (TTS) using multiple logistic regression analysis based on odds ratio (*OR*) values for criteria used to build the InterTAK diagnostic score (*top panel*). A summary score is calculated from each individual score, which facilitates differentiation of Takotsubo syndrome from acute coronary syndrome (*bottom panel*). *OR* Odds Ratio, *CI* confidence interval. (Modified and reprinted from Ghadri et al., 2017 [44] with permission)

Diagnosing TTS is often challenging, and there is currently no non-invasive tool available to facilitate conclusive diagnosis of TTS. Instead, cardiac catheterization is the method of choice to exclude or confirm TTS. The InterTAK Diagnostic Score was developed by the International Takotsubo Registry to aid physicians in assessing the likelihood of TTS (Fig. 3; [44]), but diagnostic criteria continue to be disputed (see Ghadri et al. for more details; [35]). All components of this score can be obtained in the emergency department and do not require cardiac imaging. However, without invasive assessment, a definitive diagnosis is only possible by demonstrating reversibility of the condition.

The aims of treatment for TTS are decongestion and haemodynamic support, and interventions need to be tailored to individual presentation patterns [36]. For example, in hypertensive patients with left ventricular (LV) outflow tract obstruction (as may occur with apical ballooning), arterial vasodilators must be used with caution to avoid aggravation of this complication. Guideline-directed medical therapy (GDMT) is recommended. However, no data from larger randomized trials are available regarding the efficacy of renin-angiotensin-aldosterone system (RAAS) blockers and β‑blockers in TTS patients. As outlined by a recent review, pre-existing psychiatric comorbidity requires special attention [45]. The International Takotsubo Registry demonstrated that TTS patients had significantly more depression and anxiety than ACS patients in general, even after controlling for age and sex [35]. Rapid up-titration of certain psychotropic agents (e.g. serotonin-norepinephrine reuptake inhibitors [SNRI], lithium) that tend to increase endogenous catecholamines, or electroconvulsive therapy (which also induces a transient abrupt increase in catecholamine levels), may predispose to TTS development [45]. These observations further support the concept of a causal role of excess catecholamines in this syndrome.

Long-term management strategies for psychiatric comorbidities in TTS patients, including psychotropic measures, are poorly delineated. The use of various antidepressants known to increase catecholamine levels should be avoided because of their known association with incident TTS [35]. Selective serotonin inhibitors (SSRI) may be considered to treat depression and anxiety in TTS, but there is currently a lack of data in this area [45]. However, a retrospective study of 78 patients with TTS showed increased mortality and delayed recovery of LV function in those taking SSRI [46], an observation that may caution also against the use of this substance class. Overall, there is currently no evidence of therapeutic benefit from any psychotropic therapy in TTS in the literature, and treatments that could prevent recurrence of TTS are unknown.

## Peripartum cardiomyopathy: depression and post-traumatic stress disorder

**Fig. 4:**
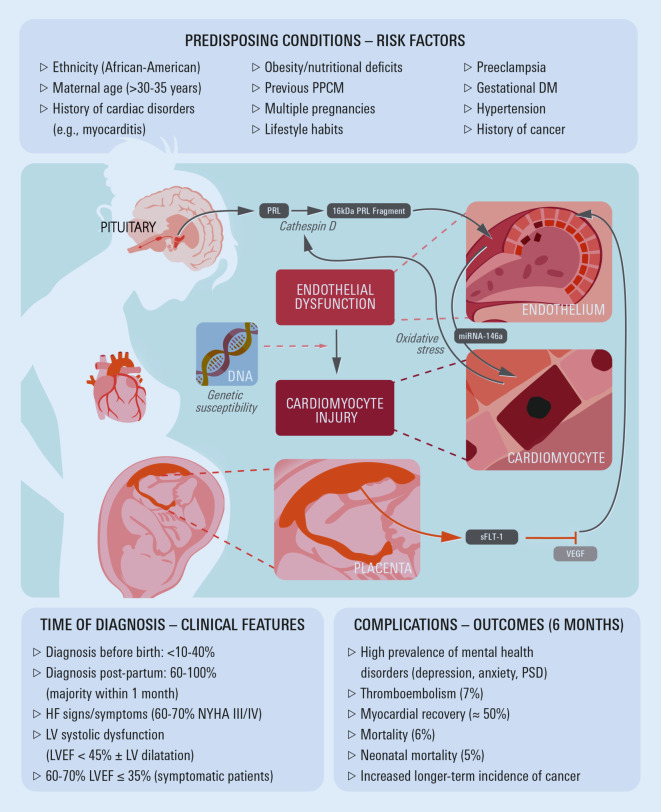
Simplified representation of possible pathogenic mechanisms of peripartum cardiomyopathy (PPCM) and predisposing conditions/risk factors. Figure design inspired by Honigberg et al., 2019 [48]. Reported 6‑month outcome data refer to Sliwa et al., 2020 [50]. *DM* diabetes mellitus, *HF* heart failure, *LV* left ventricular, *LVEF* left ventricular ejection fraction, *NYHA* New York Heart Association, *PRL* prolactin, *PSD* post-traumatic stress disorder, *sFLT‑1* fms-like tyrosine kinase receptor 1, *VEGF* vascular endothelial growth factor

Peripartum cardiomyopathy (PPCM) is an idiopathic cardiomyopathy presenting with LV systolic dysfunction (LV ejection fraction [LVEF] < 45%) with or without LV dilatation plus signs and symptoms of HF. Usually, PPCM occurs toward the end of pregnancy or in the first months after delivery. It affects about 1 in 1000 pregnancies worldwide, but incidence rates vary widely by ethnic/racial background and region; African and African American women are at a higher risk of developing PPCM. Figure 4 shows predisposing conditions and risk factors for PPCM, including, for example, ethnicity, maternal age, multiparity and pre-eclampsia. In contrast to TTS, pre-existing MHD or neurological illnesses are not among the risk factors for PPCM. Peripartum cardiomyopathy has recently been reviewed, for example, by Davis et al. [47], Honigberg et al. [48] and Bauersachs et al. [49]. The majority of women with PPCM are diagnosed after delivery, but there are large regional differences in the frequency of reported antepartum symptom onset [50]. Figure 4 details the outcomes of 739 participants in a multinational European Society of Cardiology (ESC) EURObservational Research Programme registry, in which the average 6‑month mortality rate for women with PPCM was 6%, with marked regional variability [50]. This rate is lower than previously reported [47]. Fewer than 50% of women had experienced normalization of LVEF at this time. By contrast, > 70% of a German cohort of 66 PPCM patients undergoing early treatment with the dopamine D2-receptor agonist bromocriptine and long-term GDMT had improvement in LVEF to > 50% at the 5‑year follow-up, but other cardiovascular disorders such as AF or hypertension were common [51]. A markedly increased risk for various malignancies both before and after PPCM diagnosis has been reported, possibly in association with specific genetic factors that seem to link PPCM and cancer [52].

Information on MHD following a diagnosis of PPCM is scarce. Screening for MHD with self-administered questionnaires revealed high prevalence rates of depression (32%; [53]), generalized anxiety disorders (53%; [53]) and impaired quality of life [54] in women with an established PPCM diagnosis. Using a Structured Clinical Interview, significant MHD was diagnosed in 65% of 40 women with PPCM; compared with post-partum women without PCCM, those with PCCM had a fourfold higher prevalence of major depression, a sixfold higher prevalence of panic disorder and a 14-fold higher prevalence of post-traumatic stress disorder in this study [54].

### Pathophysiology

The aetiology of PPCM is incompletely understood and likely multifactorial. Suggested, but yet unproven pathogenetic mechanisms are shown in Fig. 4. Prolactin (secreted from anterior pituitary gland) is cleaved by cathepsin D (activated by oxidative stress) into a 16-KDa fragment, which triggers endothelial dysfunction and apoptosis. Vascular damage leads to release of microRNA (mRNA)-146a and other mRNAs, which block important pathways thus inducing myocardial injury and metabolic insufficiency. Soluble fms-like tyrosine kinase receptor 1 (sFLT-1) produced by the placenta sequesters vascular endothelial growth factor (VGEF). Lack of VEGF leads to vascular and myocardial damage via several downstream pathways. Frequent occurrence of MHC is consistent with observations in severe CVD in general [5, 6]. However, in women with PPCM, impairment of the tryptophan metabolism has been reported, which increases the synthesis of quinolinic acid, with an associated reduction of serotonin synthesis, leading to reduced serotonin levels and increased production of the pro-inflammatory and MHD-promoting kynorenin, thus possibly providing a more specific explanation for the high incidence rates of MHC, especially depression, in women with PPCM [54]. This is compatible with the concept that (like certain infections) excessive psycho-physical stress may distort the interplay of the innate immune and central nervous systems, and implicates activation of toll-like receptors (e.g. TLR‑4, the transcription factor NF-kB, or the inflammasome NLRP3), as well as the secretion of interleukin‑1 beta, interleukin‑6 and other factors of the innate immune response, thus causing general symptoms of disease, but also adding to depression and anxiety [55]. Furthermore, patients with PPCM have higher levels of the depression-associated microRNA (miR)-30e [54]. Given that miR-30e impairs signalling pathways of the serotonin 1A receptor [56], it may also contribute to incident depression in women developing PPCM. Lastly, a connection between treatment with bromocriptine and MHD cannot be excluded [57].

### Management

Treatment of PPCM during pregnancy requires modifications to ensure foetal safety [47, 48]. After delivery, standard GDMT includes decongestive treatment, neurohormonal inhibitors, vasodilators and device therapies in selected patients in addition to specific pathophysiology-directed treatment with bromocriptine [58]. Improvement of HF symptoms as a result of these measures will likely also benefit the patients’ psychological situation. In view of the high prevalence of MHD in PPCM patients, psychiatric assessment and screening for post-traumatic stress disorder and other affective diseases should be part of the routine clinical evaluation. Clinical care strategies must include detailed needs analysis to facilitate personalized supportive strategies such as multimodal patient-centred management, which may also help prevent PPCM recurrence during later pregnancies. In the case of severe depression or anxiety disorders, targeted psychopharmacological and/or psychotherapeutic interventions are recommended, although systematic studies evaluating their efficacy in PPCM are lacking.

## Atrial fibrillation and cognitive decline

**Fig. 5:**
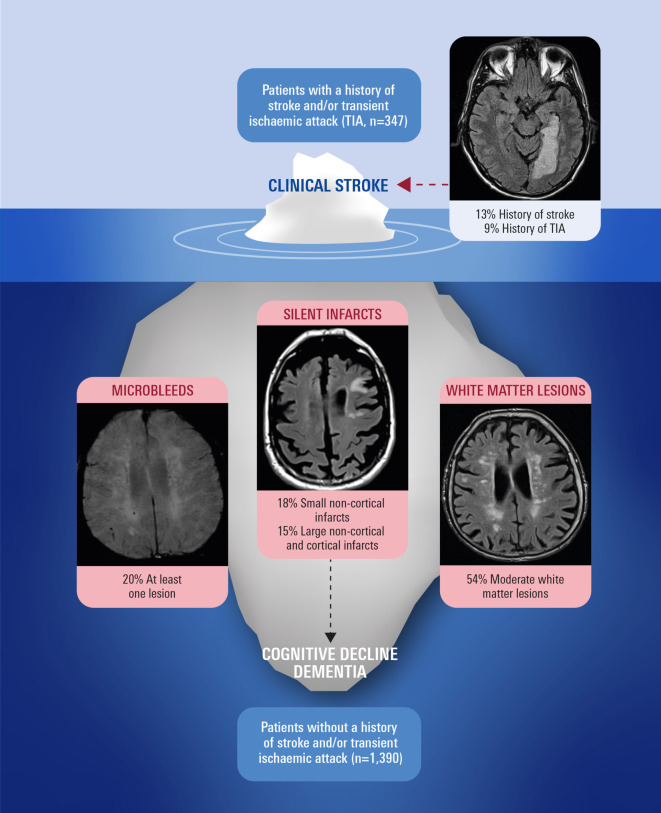
The “tip of the iceberg”. Prevalence rates of clinically apparent versus silent brain lesions in patients with atrial fibrillation (Swiss-AF prospective cohort study). (Modified and reprinted from Conen et al., 2019 [67] with permission)

Atrial fibrillation is the most common arrhythmia and its prevalence increases rapidly in aging societies [59, 60]. While relationships between AF and death, stroke and HF have long been established, more recent evidence suggests that patients with AF are also at higher risk of cognitive decline and developing dementia [61–66]. The Swiss Atrial Fibrillation Cohort Study (Swiss-AF) was a prospective, observational trial that enrolled patients aged ≥ 65 years with AF and investigated the prevalence and incidence rates of clinically apparent versus subclinical brain lesions and their association with cognitive decline over time [67]. A total of 2400 patients underwent brain magnetic resonance imaging (MRI) at baseline and after 2 years, and extensive cognitive testing was performed annually. A cross-sectional analysis from this data set revealed that clinically overt and, especially, silent brain lesions were highly prevalent (Fig. 5). For example, white matter lesions (WML) of moderate (to severe) degree were found in 54% of patients without a history of stroke or transient ischaemic attacks. The presence and volume of both overt and silent large non-cortical and/or cortical infarcts (LNCCIs) on MRI were significantly associated with reduced neurocognitive function, even after multivariable adjustment for comorbidities. The lesions could, therefore, at least partly explain a higher risk of cognitive decline in patients with AF. Remarkably, 90% of the Swiss-AF study population were on anticoagulants and/or receiving antithrombotic therapy at the time of the baseline MRI [67].

### Pathophysiology

The increased risk of cognitive impairment associated with AF seems only partly explained by the generally accepted pathophysiological concept of a causal role of thromboembolic events, including both recurrent showers of microembolism and overt strokes [62]. Previous research described a decline in cognitive function in patients with incident AF even in the absence of clinical stroke, as well as a strong relationship between the duration of AF (AF burden) and the risk of dementia in younger patients [61]. Concordantly, the Atherosclerosis Risk in Communities (ARIC) study demonstrated that patients with high AF burden (persistent AF, 100%), but not those with lower AF burden (paroxysmal AF, 1–6%), had lower executive and verbal cognitive test scores compared with patients without AF [68]. Interestingly, the observed associations remained significant after adjustment for prevalent clinical stroke and persisted even after further adjustment for subclinical events, thus not conclusively explaining the relationship between a higher AF burden and lower cognitive function. Beyond cerebrovascular embolism, clinically unrecognized (silent) strokes, microbleeds, and/or other brain lesions might have contributed to these findings [68]. In a recent Korean nationwide cohort study in AF patients treated with catheter ablation or medical therapy, ablation was associated with decreased dementia risk [69]. Notably, this relationship was also evident after censoring for stroke and adjusting for clinical confounders [69]. Together, these observations provide evidence that AF may provoke cognitive decline, and that coexistence of AF and dementia is not a chance association. However, to what extent the rhythm itself is responsible for the cognitive impairment, and to what extent eliminating the arrhythmia will diminish the likelihood of dementia requires further investigation. Other factors, such as increased systemic inflammatory activity and oxidative stress accompanying AF may also contribute to worsening cognitive function. In summary, vascular dementia appears to be a heterogenous syndrome probably caused by a variety of mechanisms, which remain poorly understood beyond the role of cortical infarcts and cerebral small-vessel disease, which is undisputed [70, 71].

Further studies addressing underlying mechanisms are underway. Thus, in Swiss-AF the serum neurofilament light chain (sNfL), an acute phase marker of neuronal damage, was found to be associated with different cardiovascular risk factors (e.g. blood pressure and diabetes) as well as with vascular brain lesions and lower cognitive function [67]. In addition, baseline sNfL predicted brain atrophy after 2 years (unpublished data). At the 2‑year follow-up, new ischaemic brain lesions had occurred on MRI in 5.5% of patients; again, the vast majority of the lesions (85%) were clinically silent and had occurred in patients on oral anticoagulation therapy. New white matter lesions were found in 18%, and microbleeds in 11%. Overall, progressive cognitive decline was found in most tested dimensions if new brain lesions were apparent on repeat MRI (unpublished data). However, to what extent these observations are causally related to the presence of AF remains to be clarified.

### Management

Whether cerebral imaging, routine neurocognitive testing and routine analysis of markers such as sNfL should be used to screen for silent brain injury and whether implementation of such strategies into clinical routine care would improve prognosis is currently unknown. Recent findings suggest that effective oral anticoagulation may at least slow down cognitive decline and the development of dementia in patients with AF [72], and elimination of AF by ablation therapy seems to reduce the risk of dementia [69]. A more thorough understanding of the pathobiology of cognitive decline and dementia in patients with this common arrhythmia is required to promote development of efficacious preventive strategies.

## Conclusion

Awareness of the pathophysiological links between cardiovascular diseases (CVD) and mental health disorders (MHD) is growing, along with better understanding of the basic mechanisms underlying their mutual impact on clinical outcomes, although significant gaps in knowledge remain. Imbalance in the interplay between the innate immune system and the central nervous system leading to systemic and cerebral inflammatory changes is emerging as a high-level player mediating the complex interactions along the heart–brain axis. Currently, MHD remain largely under-recognized in patients with CVD. This prevents comprehensive collaborative diagnosis and management of cardio-psycho-neurological disorders. Routine screening for MHD by cardiologists could help to bridge this gap and therefore offer the chance to improve patient outcomes and quality of life. Joint efforts from cardiologists, neurologists and psychiatrists are needed to advance pathophysiological knowledge and determine evidence-based treatment strategies for MHD in CVD in general. In rare diseases such as Takotsubo syndrome and peripartum cardiomyopathy, prospective randomized multicentre trials are needed to generate better evidence for the efficacy of currently applied treatments, identify (based on deeper pathophysiological understanding) more specific therapies, and better delineate targeted diagnostic and therapeutic algorithms. More comprehensive appreciation of the complex pathogenetic mechanisms of atrial fibrillation development may also lead to novel treatment opportunities to prevent brain damage and dementia in many patients with this common CVD.

## Abbreviations

ACS: Acute coronary syndrome
AF: Atrial fibrillation
CAD: Coronary artery disease
COPD: Chronic obstructive pulmonary disease
CV: Cardiovascular
CVD: Cardiovascular diseases
GDMT: Guideline-directed medical therapy
HF: Heart failure
LNCCI: Large non-cortical and/or cortical infarcts
LVEF: Left ventricular ejection fraction
MHD: Mental health disorders
MRI: Magnetic resonance imaging
NO: Nitric oxide
NYHA: New York Heart Association
PAP: Pulmonary artery pressure
PPCM: Peripartum cardiomyopathy
RAAS: Renin–angiotensin–aldosterone system
sNfL: Serum neurofilament light chain
SNRI: Serotonin-norepinephrine reuptake inhibitors
SSRI: Selective serotonin re-uptake inhibitors
TIA: Transitory ischaemic attack
TTS: Takotsubo syndrome
